# A New View of the T-Loop Junction: Implications for Self-Primed Telomere Extension, Expansion of Disease-Related Nucleotide Repeat Blocks, and Telomere Evolution

**DOI:** 10.3389/fgene.2019.00792

**Published:** 2019-08-14

**Authors:** Lubomir Tomaska, Jozef Nosek, Anirban Kar, Smaranda Willcox, Jack D. Griffith

**Affiliations:** ^1^Departments of Genetics and Biochemistry, Faculty of Natural Sciences, Comenius University in Bratislava, Bratislava, Slovakia; ^2^Lineberger Comprehensive Cancer Center, University of North Carolina at Chapel Hill, Chapel Hill, NC, United States

**Keywords:** telomere, t-loop, telomerase, replication, evolution, intron, Holliday junction

## Abstract

Telomere loops (t-loops) are formed at the ends of chromosomes in species ranging from humans to worms, plants, and with genetic manipulation, some yeast. Recent *in vitro* studies demonstrated that transcription of telomeric DNA leads to highly efficient t-loop formation. It was also shown that both DNA termini are inserted into the preceding DNA to generate a highly stable t-loop junction. Furthermore, some telomeric RNA remains present at the junction, potentially acting as a plug to further protect and stabilize the t-loop. Modeling the loop junction reveals two mechanisms by which the canonical chromosomal replication factors could extend the telomere in the absence of telomerase. One mechanism would utilize the annealed 3’ terminus as a *de novo* replication origin. *In vitro* evidence for the ability of the t-loop to prime telomere extension using the T7 replication factors is presented. A second mechanism would involve resolution of the Holliday junction present in the t-loop bubble by factors such as GEN1 to generate a rolling circle template at the extreme terminus of the telomere. This could lead to large expansions of the telomeric tract. Here, we propose that telomeres evolved as terminal elements containing long arrays of short nucleotide repeats due to the ability of such arrays to fold back into loops and self-prime their replicative extension. In this view, telomerase may have evolved later to provide a more precise mechanism of telomere maintenance. Both pathways have direct relevance to the alternative lengthening of telomeres (ALT) pathway. This view also provides a possible mechanism for the very large repeat expansions observed in nucleotide repeat diseases such as Fragile X syndrome, myotonic dystrophy, familial amyotrophic lateral sclerosis (ALS), and frontotemporal dementia (FTD). The evolution of telomeres is discussed in the framework of these models.

## Formation of T-Loops at the Telomere: A Link Between Looping and Transcription

**Figure 1:**
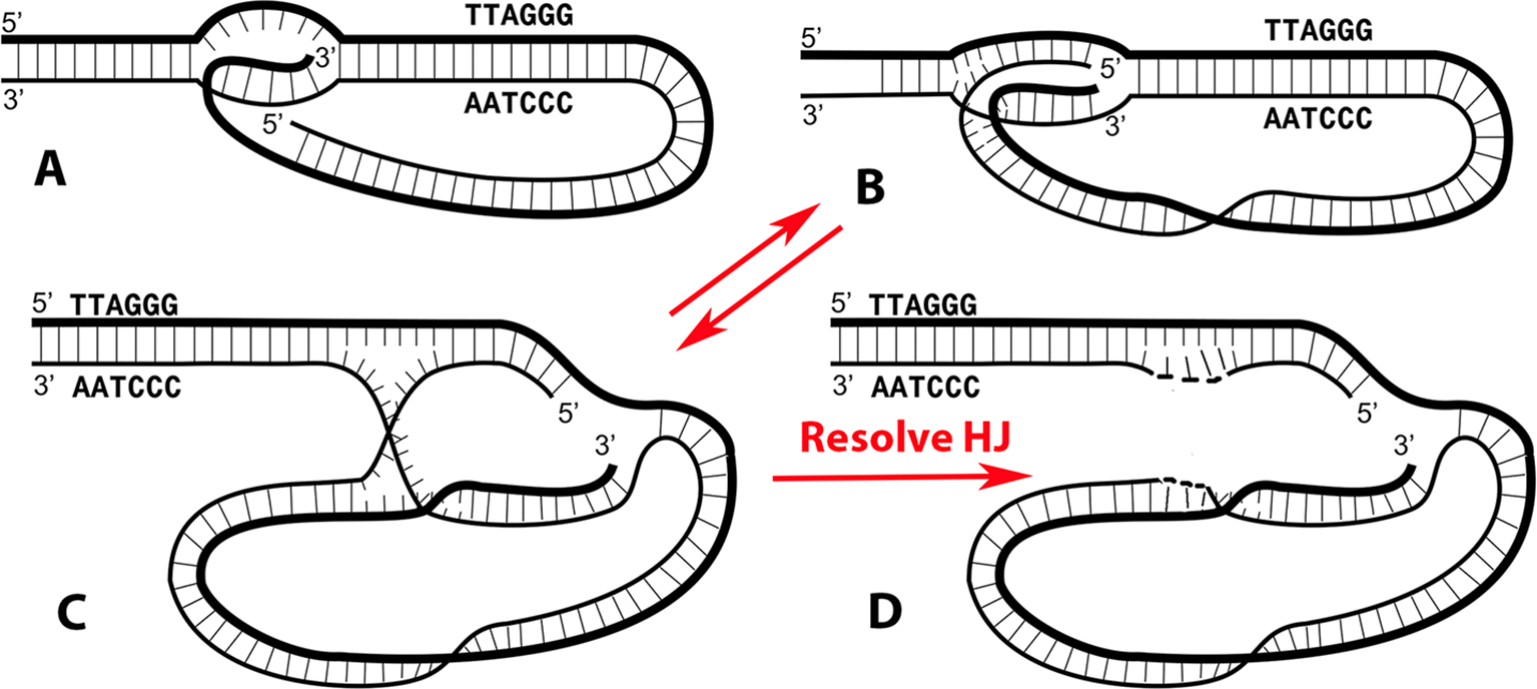
The t-loop junction contains an embedded replication origin and a Holliday junction. The classic t-loop junction **(A)** has been drawn with only the 3’ single stranded overhang from the G-rich strand annealed into the preceding DNA. In Kar et al. (2016), it was demonstrated that both terminal strands can be inserted, generating a more stable junction, illustrated with a blunt ended DNA in **(B)**. The structures in both A and B contain an embedded 3’ terminus capable of acting as a replication origin. The structure in **(B)** is topologically equivalent to that shown in **(C)**, illustrating the presence of a classic Holliday junction. Resolution of the Holliday junction by resolvases such as GEN1 or SLX1/4 will generate a rolling circle replication template **(D)**. Panels **A–C** are modified from Kar et al. (2016).

Looping the end of the telomere back into the preceding (5’-TTAGGG-3’)_n_ repeat sequence to generate a lasso-like structure (t-loop) was first described in 1999 (Griffith et al., 1999) in human and murine cells, and in following studies extended by us and others to trypanosomes, *Oxytricha*, chickens, *Caenorhabditis elegans*, plants, and with some genetic manipulation, to yeasts [reviewed in Doksani et al. (2013) and Griffith (2013)]. These studies employed electron microscopy (EM) to directly visualize the DNA loops. A major stride was accomplished in 2013 when Doksani et al. (2013) visualized t-loops using stochastic optical reconstruction microscopy (STORM) imaging and employed this technique to confirm the presence of t-loops in murine cells and demonstrate their dependence on functional TRF2 protein. The rationale which drove the original proposal of t-loops was based on our knowledge of the mechanism of homologous recombination (HR) and the fact that a double-stranded (ds) DNA containing a long single-stranded (ss) overhang with a 3’ terminus provides a highly efficient template for strand invasion of the ssDNA into a homologous dsDNA in reactions driven by proteins such as recA, uvsX, or Rad51. The telomere represents a structure in which both pairing partners are present in the same molecule. The demonstration of t-loops and this thinking led to the classic diagram of a t-loop (**Figure 1A**) in which the bubble formed at the t-loop junction contains just the 3’ ssDNA overhang inserted. However, even in early papers, it was proposed that the 5’ strand (Tomaska et al., 2002) or both strands may also insert, leading to a more stable t-loop junction (Stansel et al., 2001).

The discovery that telomeres are transcribed (Azzalin et al., 2007; Schoeftner and Blasco, 2008) and that the G-rich RNA termed TERRA is increased in cells with elevated HR (Arora et al., 2014) led us to ask if t-loop formation might be related to TERRA formation. In Kar et al. (2016), we developed a simple *in vitro* system in which a terminal block of 576 bp of TTAGGG repeats in a linearized plasmid can be transcribed through the repeats by T7 RNA polymerase to yield TERRA. Following transcription, EM was used to probe for the presence of t-loops at the end of the DNA containing the mini-telomere. It was found that transcription of the telomeric block led to highly efficient t-loop formation with up to 60% of the input DNA containing a 576-bp or smaller loop at the telomeric end. Surprisingly, t-loops were formed at high levels irrespective of the presence of a long 3’ ss overhang. Telomeres that were blunt ended, contained a 4-nt 5’ overhang or 3’ overhangs of 54–96 nt presented equally good templates for looping. Examination of the t-loop junction showed the frequent presence of a small nucleic acid bead, which, when labeled with a biotin-tagged RNA precursor, revealed that some TERRA remained at the junction in spite of RNase treatment. The results forced a revision of the t-loop junction model.

## Consequences of a T-Loop Junction with Both Termini Inserted

 In the new t-loop model (**Figure 1B**), both terminal strands are annealed to their complementary strands in the bubble. This structure has features of both a replication fork (right side) and a Holliday junction (left side). With both strands base paired, the t-loops would be much more stable than with just the ss overhang paired. Although comparison of the stability of single- versus double-stranded paired t-loops will need to be quantified by assessment of their thermodynamic properties (such as free Gibbs energy), we have observed that t-loops generated by transcription in vitro were highly stable to deproteinization and remained present for days at 4°C (Kar et al., 2016). The suggestion that the 3’ G-rich overhang is not essential for t-loop formation is in line with the observation that while t-loops have been seen in plants (Cesare et al., 2003) and can be very large, it remains unclear whether significant ss overhangs are present at plant telomeres. Furthermore, in worms, some telomeric termini contain 5’ ss termini (Raices et al., 2008). Based on these new findings, a model of t-loop formation was proposed by Kar et al. (2016) in which transcription of the telomere opens the DNA helix, frequently leaving R-loops behind. These would provide preferential sites for shelterin complexes (Palm and de Lange, 2008; Erdel et al., 2017) loaded at the telomeric terminus to loop back and establish a stable t-loop. The central function of the long 3’ ss overhang in mammalian telomeres may be loading Pot1 and the shelterins at the end of the telomere enabling them to interact with other shelterin complexes bound to internal R-loops along the telomere. Indeed, TERRA has been shown to bind TRF2 (Deng et al., 2009) and thus would recruit a shelterin complex to an internal R-loop. Such interactions would generate a broad distribution of sizes for the circular portion of the t-loop, from large loops formed near the subtelomeric repeats to smaller loops formed at sites closer to the telomere end. This is, in fact, what is seen in naturally isolated t-loops (Griffith et al., 1999; Doksani et al., 2013).

In early t-loop studies (Griffith et al., 1999; Stansel et al., 2001), it was pointed out that a 3’ ss telomeric overhang base paired in the t-loop bubble presents a *de novo* replication origin. This remains so with the newer model (**Figure 1B**). Self-primed telomere extension from the t-loop would employ the normal cadre of chromosomal replication factors including a DNA polymerase, sliding clamp, helicase, and primase among others. The kind of telomeric extension exemplified by this experiment has features of the alternative lengthening of telomeres (ALT) phenomenon. Telomeres in ALT cells (which lack active telomerase) show a much broader range of sizes and changes in size than normal cells (Bryan et al., 1995), reviewed in (Cesare and Reddel, 2010). In self-primed replication, the degree of extension will vary from short (small terminal t-loop) to long (large t-loop with the bubble close to the sub-telomeric sequences). Extension from the annealed 3’ terminus will generate a ssDNA loop as replication proceeds toward the end of the telomere followed by its release as a longer ss tail upon encountering the telomere end. Conversion to duplex DNA would be carried out by the chromosomal replication factors.

*In vivo*, access of the chromosomal replication factors to the embedded 3’ telomeric terminus in the t-loop must be tightly controlled. Otherwise, telomeric extension *via* self-priming could go on unchecked in a cellular environment where telomerase would be expected to provide a more precise control over telomere length. Access could be blocked by assembly of the shelterin complexes at the t-loop junction. In addition, in Kar et al. (2016), we showed that a visible particle containing TERRA was often present at the t-loop junction. This RNA very likely was present in a G-quadruplex-stabilized structure formed between TERRA and the G-rich telomeric strand. It was relatively resistant to several RNases including RNase H and H1. While the detailed organization of the t-loop bubble containing TERRA remains to be determined, the presence of an “RNA plug” may further stabilize the t-loop structure and sequester it from unwanted activation. Further analysis using the *in vitro* system may shed light on these possibilities.

**Figure 2:**
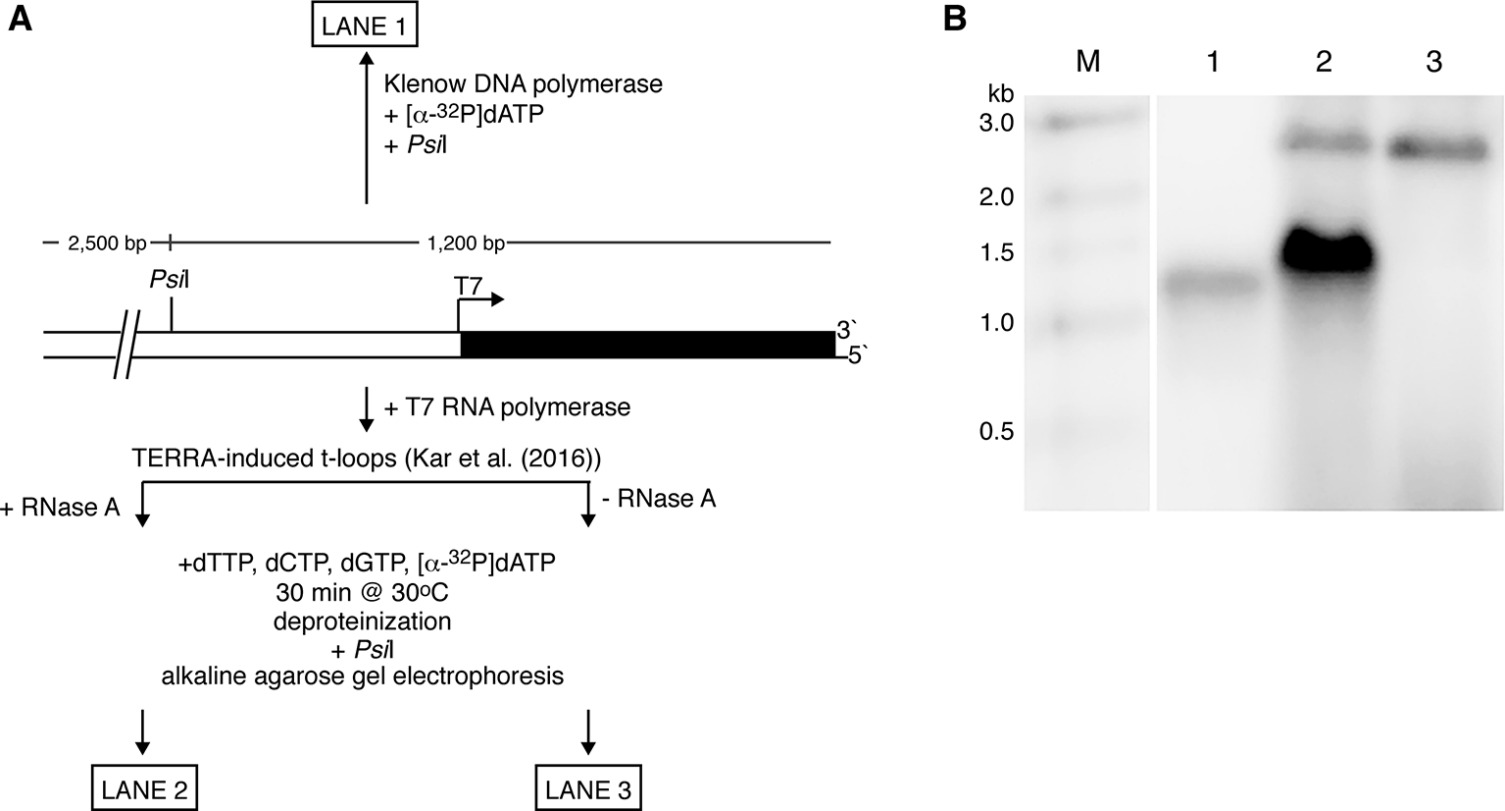
Self-primed telomere extension from a t-loop. **(A)** Experimental strategy. The plasmid pRST5 linearized with *Bsm*BI contains a block of 576 bp of (TTAGGG)_n_ repeats at one end and terminates with a 4-nt ss overhang at the 5’ end. Transcription by T7 RNA polymerase begins at a promoter located at the junction of the plasmid and telomeric repeats and moves through the repeats to the DNA end-producing TERRA RNA (G-rich transcript) as described in Kar et al. (2016). This also generates a high fraction of the DNA with the telomeric tract arranged into t-loops. **(B)** If the linear pRST5 DNA is labeled at the 3’ telomeric terminus with [α-^32^P]dATP and then cleaved with *Psi*I, a 1,200-nt labeled fragment is seen on an alkaline denaturing gel (lane 1). Linear pRST5 DNA (1 µg) arranged into t-loops by transcription and treated with RNase A was mixed in a buffer containing 10 mM HEPES–NaOH pH 7.5, 50 mM NaCl, 10 mM MgCl_2_, 1 mM EDTA, and 2 mM 2-mercaptoethanol. The reactions also contained 0.3 mM ATP and CTP, and 0.6 mM dTTP, dCTP, and dGTP. To this mixture was added 0.5 µl of 10 mCi/ml [α-^32^P]dATP. The T7 replication factors were then added (T7 DNA polymerase with thioredoxin 1 µl of 200 ng/µl; T7 gene 4 protein 0.4 µl of 6 µM; and T7 gene 2.5 protein 1 µl of 1 mg/ml) and the mixture incubated for 30 min at 30°C. Following incubation, the DNA was deproteinized with SDS and proteinase K, treated with *Psi*I, then denatured and electrophoresed on the alkaline gel (lane 2). When the DNA was taken through the same steps as in lane 2 but had not been treated with RNase A, no incorporation in the range between 1,000 and 2,000 nt was observed (lane 3). The higher bands in lanes 2 and 3 represent some of the larger 2.5-kb *Psi*I plasmid fragment, which had acquired label at the distal end containing a 5’ overhang. Lane M shows molecular weight markers.

To conduct a “proof of principle” test of the ability of a t-loop to self-prime telomeric replication, we utilized the T7 phage replication factors and linear pRST5 plasmid DNA containing t-loops generated by T7 RNA polymerase transcription through the TTAGGG repeats as described in Kar et al. (2016). The T7 factors include T7 gene 5 DNA polymerase with thioredoxin, gene 4 helicase–primase, and gene 2.5 single-strand binding protein (Kulczyk and Richardson, 2016). Treatment of the linear DNA with *Psi*I endonuclease generates a 1.2-kb fragment containing the telomeric block and a larger 2.5-kb plasmid fragment. The DNA (not arranged into t-loops) can be labeled at the telomeric 3’ terminus with Klenow DNA polymerase and [α-^32^P]dATP in an exchange reaction. Subsequent cleavage with *Psi*I and electrophoresis on a denaturing alkaline gel results in a ssDNA band at the position of a 1,200-nt fragment (**Figure 2**, lane 1). To ask if the 3’ terminus embedded in a t-loop can self-prime extension by the T7 replication factors, the 3.5-kb plasmid arranged into t-loops (including deproteinization and RNase A treatment to remove RNA at the t-loop) was incubated with the T7 replication proteins for 30 min at 30°C (see **Figure 2** legend for details) in a reaction containing [α-^32^P]dATP. This was followed by cleavage with *Psi*I and electrophoresis on an alkaline gel. A strong band of label incorporation (lane 2) into DNA was observed with the median position shifted to a molecular weight higher than the control band in lane 1. Depending on the size of the t-loop formed at the end of the pRST5 DNA, self-primed extension could increase the size of the *Psi*I fragment by an amount between ∼100 (the smallest t-loop observed) and ∼550 nucleotides (the largest) resulting in a distribution of fragments on the gel between 1,300 and 1,750 nucleotides. This is what was observed. We also found that removal of the RNA by stringent RNase A treatment was absolutely required to observe self-primed replication. Shown in lane 3 is the result of an experiment identical to that in lane 2 except that the DNA was not treated with RNase A following transcription and deproteinization. This results in an abundance of RNA remaining along the telomeric tract [see **Figure 3A** in Kar et al. (2016)]. In this case, no incorporation of radiolabel was observed following incubation with the T7 proteins (lane 3) showing that the presence of TERRA strongly inhibited self-primed extension. The higher bands in lanes 2 and 3 represent the larger *Psi*I fragment, which has acquired some label. Of note, DNAs not arranged into t-loops would not be expected to incorporate label as they would lack the embedded 3’ self-priming terminus and in agreement, little or no label was seen in lane 3 at the position of the nonextended fragment (lane 1). Clearly, further work would employ a fully reconstituted eukaryotic replication system, such as those that have been developed in *Saccharomyces cerevisiae* (Yeeles et al., 2015), and larger t-loop substrates.

**Figure 3:**
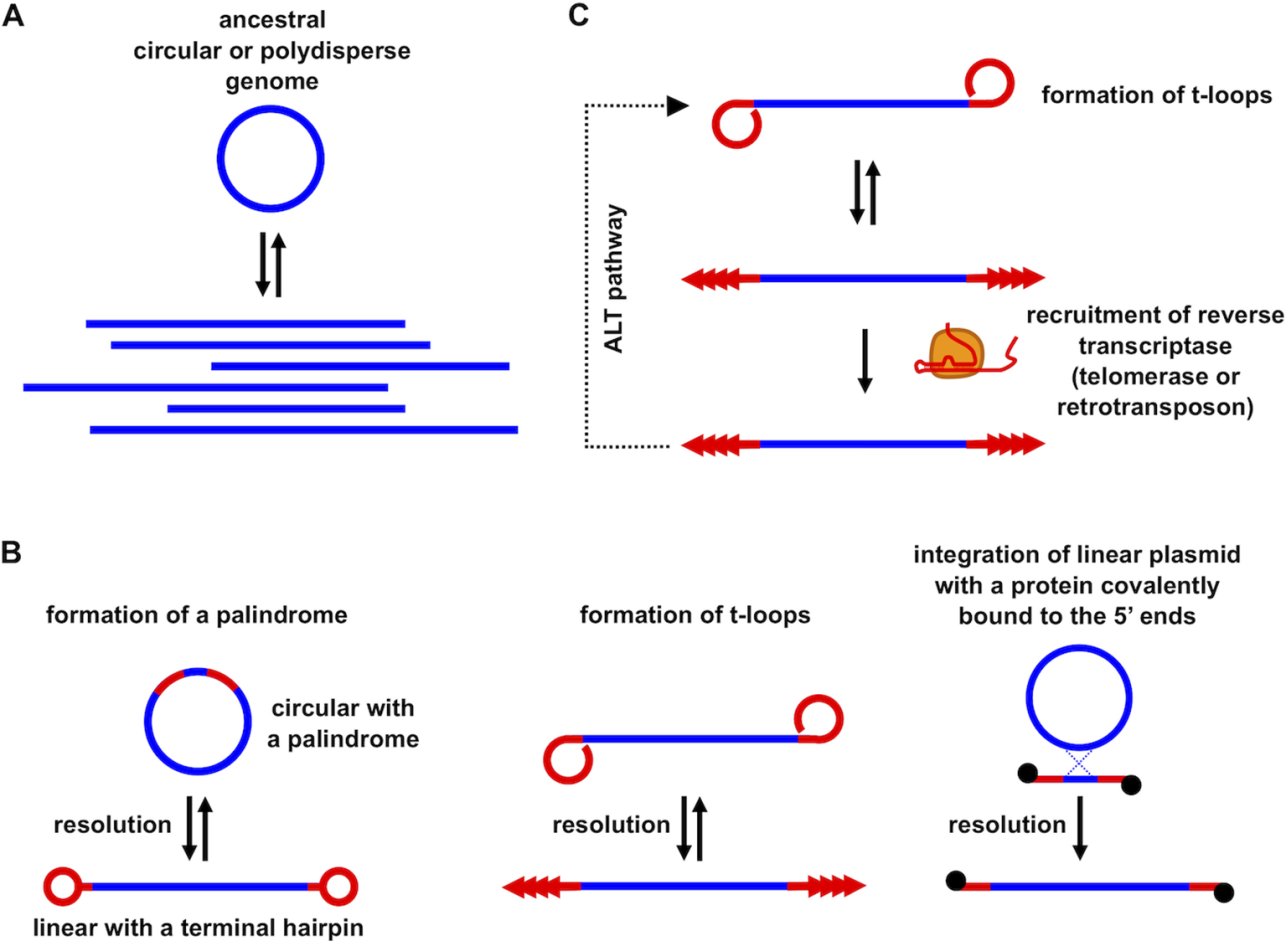
The role of t-loops in the evolution of linear eukaryotic chromosomes and telomeric repeats. **(A)** Linear DNA molecules of heterogeneous lengths with a variety of branched structures (i.e. polydisperse DNA) present in most prokaryotes may correspond to an ancestral state in the early stages of eukaryotic chromosome evolution; **(B)** linear chromosomes terminating with specific telomeric structures might have emerged by applying various molecular mechanisms (e.g., by resolution of a palindromic repeat into terminal covalently closed hairpins, t-loop driven replication generating tandem repeat arrays, recombination with plasmid or viral linear DNA possessing a protein covalently bound to the 5’ termini); **(C)** replacement of t-loop based telomere maintenance by recruitment of retrotransposon-derived RT and/or telomerase and retaining t-loops as an ALT pathway.

## Conversion of the T-Loop to a Rolling Circle Template

Inspection of the new model of the t-loop bubble (**Figure 1C**) reveals that resolution of the Holliday junction (HJ) at the point where the two ssDNAs cross over each other will generate a covalently closed ssDNA circle annealed to the strand containing the free 3’ terminus (**Figure 1D**). HJ resolution at the t-loop thus creates a classic rolling circle template capable of extending the preceding DNA by large amounts. This would employ the canonical chromosomal replication factors. In human cells, resolution could be accomplished by GEN1 or the SLXC1/4-MUS81 complex (Rass et al., 2010; Garner et al., 2013; Wyatt et al., 2013; Wyatt and West, 2014; Chan and West, 2015). This presents a second pathway by which t-loops can function to extend the telomere.

Probably, the greatest challenge will be to show that t-loops corresponding to the structures depicted by **Figures 1B, C** are formed *in vivo*. One possible experimental approach would be based on the *in vitro* observation that t-loops are induced by transcription and stabilized by TERRA. So far, telomeric fragments have been isolated from cells as either deproteinized DNA or as DNA–protein complexes (disregarding RNA). It would be worth trying to purify telomeric fragments associated with TERRA from cell nuclei. This could be done either by immunoprecipitating one of the shelterin components under RNase-free conditions or by directly purifying TERRA in complex with telomeric DNA. The latter approach could be based on the CHIRT protocol developed to investigate the genomic binding sites of TERRA transcripts (Chu et al., 2017). The topology of the DNA–TERRA complexes would then be assessed by means of electron microscopy combined with mapping the double- and single-stranded regions.

## Nucleotide Repeat Expansion: Can This Mechanism Explain the Very Long Expansions Seen in Some of the Nucleotide Repeat Diseases?

In the classic nucleotide repeat diseases, short blocks of repeating tri- to hexa-nucleotides which are normally stable in the genome expand in size with consequences resulting in severe disease. In Huntington’s disease, (CTG)n expansions in the coding sequences for the huntingtin protein are relatively modest, growing from ∼10 to 20 repeats in the unaffected genome, to ∼100 repeats in severely afflicted individuals [reviewed in Bates et al. (2015)]. However, very large expansions are observed in other diseases. In Fragile X syndrome, the normal repeat block of 10–25 (CCG)n triplets may expand to over 1,000 bp in afflicted male patients [reviewed in Ciaccio et al. (2017)]. The myotonic dystrophy type 2 locus begins with a (CCTG)n repeat block a few tens of repeats in size but can grow to thousands of base pairs in patients presenting with the disease (Liquori et al., 2001). More recently, in familial amyotrophic lateral sclerosis (ALS) and frontotemporal dementia (FTD), a hexanucleotide block (CCGGGG)n in the C9orf72 locus which is normally a few tens of repeats in size has been shown to expand to thousands of base pairs (DeJesus-Hernandez et al., 2011; Renton et al., 2011).

The molecular mechanisms by which expansions are initiated and grow have been the topic of many studies but to date are not fully understood. The predominant model poses that replication through the repeat block results in backwards slippage of the polymerase machinery and looping out of one of the strands (Wells and Ashizawa, 2006). If the ssDNA in the loop can pair on itself as in the (CTG)_n_ or (CCG)_n_ repeats, this will produce a partially base paired stable hairpin at the fork. Nicking and filling in steps would then add the looped-out nucleotides to the repeat block. However, ssDNA with the (GAA)n repeat that expands in Friedreich’s ataxia cannot form partially paired hairpins. More importantly, the very large expansions seem difficult to explain by simple slippage models since this would generate extremely large ssDNA loops at the replication fork if expansion were to occur in one event and these would be targets for nucleases. Hence, it seems possible that the very large expansions involve a two-step mechanism possibly beginning with replication slippage but then employing a different mechanism in the second stage potentially involving the formation of a t-loop like structure.

The models described above offer possible mechanisms for the very large nucleotide repeat expansions. Replication through long (5’-CCGGG-3’)_n_ repeats can result in replication fork stalling (Thys and Wang, 2015) producing ds breaks. A break within the nucleotide repeats would create two DNA ends, each with similarities to telomeres and with the capability of forming “t-loops” either spontaneously, following transcription through the DNA to the ends, or by action of HR factors including Rad51. Once formed, the loops would protect the broken ends from recognition as ds breaks. However, they could also generate cycles of self-primed replicative extension either by initiation from the embedded 3’ terminus or *via* HJ cleavage and production of a rolling circle template. These expansions would eventually be followed by opening the loops and fusion of the repeat block, now expanded greatly by the cycles of self-primed replication, back into the chromosome.

## Evolutionary Implications

The involvement of t-loops in DNA transactions described above supports the participation of these structures in the evolution of telomeres. The origin of linear chromosomes and telomeres providing solutions to end-protection and end-replication problems are enigmatic and still a matter of debate (e.g., Nakamura and Cech, 1998; Ishikawa and Naito, 1999; de Lange, 2004; Fajkus et al., 2005; Nosek et al., 2006; Linger and Price, 2009; Lue, 2010; Forterre, 2013; Garavís et al., 2013; Fulcher et al., 2014; de Lange, 2015; Slijepcevic, 2016; Cervenak et al., 2017; Lue, 2018). Even the basic question regarding the advantage of linear versus circular genomes in eukaryotes remains unanswered. The early speculation supported by the results from mutants of fission yeast *Schizosaccharomyces pombe* possessing circularized chromosomes, i.e., that circular chromosomes would compromise meiotic segregation (Ishikawa and Naito, 1999), was questioned (de Lange, 2015) by the existence of the bacterial XerD/C resolution machinery efficiently cleaving circular chromosomes at specific *dif* sites (Barre et al., 2001). Furthermore, many eukaryotes lack sexual reproduction and do not undergo meiosis, yet they retain linear chromosomes and canonical telomeres. These arguments support the conclusion that telomeres are structures exhibiting very potent means for escaping recircularization through ligation (de Lange, 2015), and t-loops provide a simple, powerful means of blocking circularization. In addition, it has been proposed that centromeres were derived from telomeres during the evolution of eukaryotic chromosomes implying that linear chromosomes arose even before the emergence of a mitotic segregation machinery (Villasante et al., 2007). Another line of speculation would be that large eukaryotic genomes are better off being fragmented into a number of linear chromosomes whose ends would provide the possibility of their specific spatial organization/localization and/or means for transcriptional regulation of genes. However, the reduction in the number of chromosomes in *S. cerevisiae* from 16 to 1 or 2 did not yield any substantial changes in the transcriptomes of such strains compared to the wild type (Luo et al., 2018; Shao et al., 2018). In addition, the existence of an ant species possessing a single chromosome demonstrates that even a complex eukaryote does not need to have a fragmented genome (Crosland and Crozier, 1986). All in all, these results suggest that linearity of eukaryotic chromosomes might have been forced by a selfish element(s) without necessarily providing selective advantage for the host.

In line with this hypothesis is the scenario that takes into account early events in eukaryogenesis (de Lange, 2015). It was indicated that accommodation of a α-proteobacterial endosymbiont that gave rise to mitochondria was accompanied by a massive invasion of group II introns (Martin and Koonin, 2006). According to de Lange (2015), the spreading of introns across the genome resulted in the inevitability of linear chromosomes. The occurrence of a double-strand DNA break would be either repaired by nonhomologous end joining pathways, leading to recircularization of the genome, or through HR resulting in the formation of a loop *via* invasion of the broken end into a copy of the intron. When the number of introns was low, the HR pathway would result in repair of the double-strand break followed by recircularization of the chromosome. However, as the number of introns increased, the chance of recircularization of a chromosome decreased, since it would require the two loops present at the ends of a single DNA molecule to be passed by replication forks synchronously. Under these circumstances, the t-loops would provide solutions for both end-protection and end-replication problems serving as primordial telomeres, later replaced by a more robust telomerase-dependent telomere replication (de Lange, 2004; de Lange, 2015).

This appealing scenario also implies that the t-loop-based telomere formed on intronic repeats was replaced by modern telomeres by accommodation of a telomerase reverse transcriptase derived from a reverse transcriptase (RT) encoded by group II introns that gained the ability to use the 3’ end of a chromosome as a primer for reverse transcription of its associated RNA, derived from another group II intron. This would be followed by the development of specific interactions between RT and RNA, the reduction in the RNA template region to a short sequence that will eventually become a terminal repeat and by recognition of all chromosomal ends by the same RT/RNA pair.

Although this is a plausible chain of events, the hypothesis of eukaryogenesis based on a massive invasion of group II introns has its opponents. For example, Poole (2006) argues that whereas selfish genetic elements (i.e., group II introns) are tolerated by sexual organisms, this is not the case for asexual species. Moreover, group II introns were repeatedly introduced into Archaea (i.e., Euryarcheota), apparently without massive expansion accompanied by linearization of their genomes (Simon et al., 2008). Hence, massive expansion of these introns might have occurred later in evolution and could be associated with increased host fitness, as it has been shown that spliceosomal introns play a role in survival of yeast cells during starvation (Morgan et al., 2019; Parenteau et al., 2019).

In addition to these observations that do not comply with the role of group II introns in the evolutionary emergence of linear chromosomes, the scenario proposed by de Lange (2015) implies that the evolution of telomeres must have proceeded in multiple steps and thus would require a substantial time. Why then are telomeres so conserved in such diverse eukaryotes as protists, fungi, plants, and animals [assuming that the exemptions like retrotransposons in *Drosophila* (Pardue and DeBaryshe, 2008), large tandem repeats in *Chironomus*, or extremely variable telomeric repeats in many yeast species (Lue, 2010; Cervenak et al., 2017) are secondary derivatives of the canonical TTAGGG-like repeats maintained by telomerase]?

If we disregard the possibility of a convergent evolution of modern telomeres that in independent phylogenetic branches yielded the same result, there are several possible scenarios. First, the early evolution of eukaryotes could have taken place in a distinct ecological niche for a period of time sufficient to slowly replace the ancestral telomeres [e.g., those depicted by de Lange (2015)] with their modern counterparts in a small population of cells representing the Last Universal Eukaryotic Ancestor (LECA) that subsequently spread around the globe. Second, telomerase could have been present in the pre-eukaryotic host cell (e.g., Nakamura and Cech, 1998) before acquisition of the α-proteobacterial symbiont and thus was preadapted for rapid complementation of the t-loop system of telomere maintenance. This would be in line with the hypothesis of Cavalier-Smith (2010) stating that linear chromosomes with telomeres existed before the endosymbiotic event leading to the origin of the eukaryotic cell. However, there is no report of linear bacterial or archaeal chromosomes containing telomerase-derived arrays at the ends, so this hypothesis is not supported by solid evidence.

A third scenario assumes that it might have been the other way around (**Figure 3**). As argued for a long time by Arnold Bendich, the genomes of prokaryotes, even though they exhibit circular physical maps (e.g., by restriction enzyme mapping or sequencing), are in fact linear and polydisperse *in vivo* (Bendich, 2001). In contrast to “true” linear genomes such as chromosomes found in eukaryotic nuclei, they do not possess telomeres and thus are easy targets of DNA-damage response machinery and other DNA processing enzymes. The polyploid nature of prokaryotic genomes (Soppa, 2014) makes them suitable for evolutionary experimentation including tinkering with their topology. Indeed, there are numerous examples of Archaeal linear extragenomic genetic elements employing various strategies of maintaining their ends (Pina et al., 2014; Wang et al., 2015). It is conceivable that the pre-eukaryotic ancestor stabilized the ends through a protective structure similar to that present in several species of bacteria [e.g., *Agrobacterium*, *Borrelia*, *Coxiella*, *Streptomyces* (Volff and Altenbuchner, 2000)] or mitochondria in numerous modern eukaryotes (Nosek and Tomaska, 2003; Smith and Keeling, 2015). These examples illustrate that formation of a telomere was not a eukaryotic innovation but could occur quite frequently in prokaryotes. Although linear prokaryotic and organellar genomes employ several types of strategies of how to stabilize their DNA ends, one of the most similar to eukaryotic telomeres is based on very diverse sequences whose only common feature is the ability to replicate autonomously *via* a rolling circle mechanism generating a long array of tandem repeats (Nosek et al., 2005; Nosek et al., 2006; Tomaska et al., 2009). Autonomously replicating circular DNA molecules undergoing frequent excision–integration cycles enabling their expansion were observed in a wide range of cells (Cohen et al., 2007; Møller et al., 2015; Shoura et al., 2017), and may lead to a formation of tandem repeat arrays. If such arrays are integrated at the unprotected end of a DNA molecule, it would immediately provide a substrate for the formation of a t-loop, thus solving the end-protection and end-replication problem in a single step [for more details see, Nosek et al. (2006)]. Moreover, the tandem repeat sequences could undergo transcription, generating a population of RNA molecules similar to TERRA. These, as outlined above, could even pronounce the propensity of the sequence to form t-loops (Kar et al., 2016). After such a host cell engulfed the future mitochondrion, the invasion of group II introns would initiate eukaryogenesis (Martin and Koonin, 2006) and at the same time provide candidate RTs for future telomerase development. The only step required to transform an RT to a modern-type telomerase would be acquiring the ability to associate with RNAs derived from preexisting telomeres and employ a small sequence as a template and the rest of the molecule as a structural scaffold. The template sequence might have been short and conserved because it provided some particular structural features (e.g., formation of a protective secondary structure such as the G-quadruplex), whereas the rest of the RNA molecule, as well as the proteins associated with telomeres and telomerase were much more prone to evolutionary changes (e.g., Fajkus et al., 2005; Linger and Price, 2009; Lue, 2010; Fulcher et al., 2014; Cervenak et al., 2017). Retainment of t-loops as a backup or complementary means for solution of end-protection and end-replication problems was dependent on the evolutionary trajectory of a particular phylogenetic line. For example, in macronuclei of ciliates with very short telomeric tracts, or in yeast species with short (300–500 bp) telomeric tracts containing heterogeneous repeats (e.g., *S. cerevisiae*, *S. pombe*), t-loops are difficult to form even from a mechanistic point of view (e.g., limitations associated with DNA bending), and their absence is compensated by other mechanisms such as amplification of subtelomeric regions, gene conversion, or HR [reviewed in Wellinger and Zakian (2012)]. However, even these organisms retained their capacity to form t-loops as exemplified by their observation in micronuclei of *Oxytricha* (Murti and Prescott, 1999) or the ability of *S. pombe* telomeric protein Taz1 to promote formation of t-loops *in vitro* (Tomaska et al., 2004).

In summary, the recent results indicating the participation of t-loops in various forms of DNA transactions shed additional light not only on the maintenance of telomeres but also has much broader implications ranging from the molecular basis of nucleotide repeat expansion to the evolution of telomeres.

## Author Contributions

AK carried out the replication experiment. JDG, JN, and LT developed the hypotheses and wrote the original draft. JDG, LT, JN, and SW prepared the later and final drafts of the work. JDG, AK, LT, and JN prepared the figures. All authors approved of the final work.

## Funding

This work was supported by grants from the NIH (GM31819, ES013773) to JDG and APVV-15-0022 and VEGA 1/0052/16 (to LT) and APVV-14-0253 and VEGA 1/0027/19 (to JN).

## Conflict of Interest Statement

The authors declare that the research was conducted in the absence of any commercial or financial relationships that could be construed as a potential conflict of interest.
